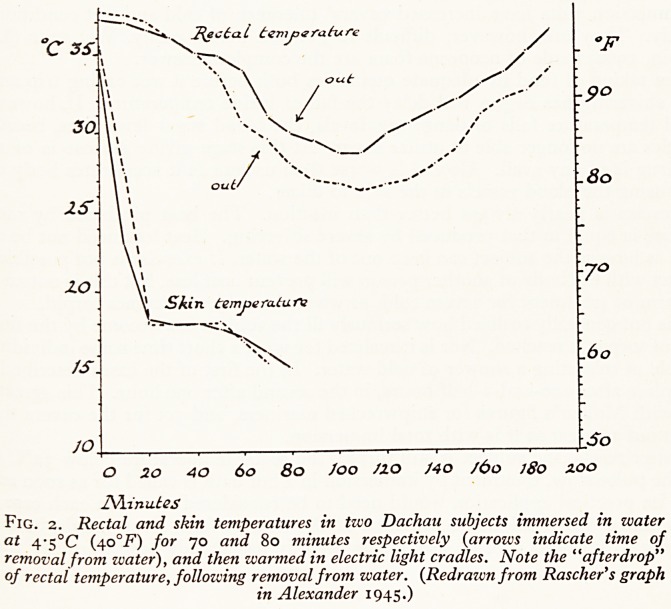# Cavers Dying of Cold
*This paper was read to the Summer Meeting of the Cave Research Group of Great Britain at Brecon, on the 29th June 1963.


**Published:** 1964-01

**Authors:** Oliver C. Lloyd

**Affiliations:** Department of Pathology, University of Bristol


### CAVERS DYING OF COLD*

BY

OLIVER C. LLOYD

*Department of Pathology, University of Bristol*

There have been two fatal accidents to cavers on Mendip due to cold. It is the purpose of this paper to describe them briefly, to say what is known about the effect of cold on the human body and to outline principles of prevention and treatment.

### CASE REPORTS

*Case 1.*

J. W. was a young man of 23, who had been caving occasionally for two years, but was inexperienced. On Saturday 17th January 1959, he went down Swildon's Hole in company with a great many others to help as a porter on a diving expedition to Swildon's IV, descending the cave at about noon. At 3.30 p.m. the cave began to flood. Diving was abandoned and at 7.30 p.m. J. W., tired, cold and inadequately clad for such conditions, tried unsuccessfully to climb the flooded 40 ft. pot. He was with difficulty hauled up on a line, and became unconscious while the water was pouring onto him. At the head of the pitch he recovered consciousness and was able to stand up with support, and to eat some chocolate, but he was unable to converse. He was very cold. The temperature of the water was not measured but was likely to have been about 40°F (4·5°C). While he was being taken out of the cave he died suddenly at about 9 p.m., that is one-and-a-half hours after his condition had first given rise to alarm. J. W. did not have a robust constitution and was likely to faint, if he was hurt.

Post-mortem examination performed by Dr. R. L. Bishton the next day showed death to have been due to acute heart failure, with extreme dilatation of the right auricle and ventricle and of the great veins which enter them. The lungs were dry. The chocolate had reached the stomach.

*Case 2.*

H. M. was a thin, healthy young woman of 17. On Sunday 17th March 1963, she went down Longwood Swallet with a party from her college. It was her first caving trip. They entered the cave at 1.45 p.m. when it was raining heavily, and by 2.45 p.m. they had all got soaked through and turned back. She was only lightly clad. There was considerable delay at the 10 ft. overhang, because a member of one of the parties in front had sprained his ankle and was slow to get out. Everyone got cold but not uncomfortably so, and when H. M. went through the S-bend at about 4.20 p.m. she had no difficulty. By now the water was pouring down the entrance shaft (which is a narrow vertical, 40 ft. high) and she was getting drenched, as there was no shelter. The temperature of the water was not measured, but was probably about 43°F (6°C). At 4.30 p.m. she tried to climb the ladder in the entrance shaft but failed. She was by now frightened. At 4.45 p.m. the Mendip Rescue Organisation was called out. At 5.15 p.m. she was found by an advance party to be in a state of stupor, unconscious, curled up, groaning. At 5.30 p.m. she was offered some hot soup but was not only unable to drink it; she did not even feel it burn her lips. Some was poured into her mouth and entered her larynx. It is evident that she was already dead. Only one hour had elapsed since her condition first gave rise to alarm.

Post-mortem examination performed by Dr. D. H. Johnson the next day showed that death was due to acute heart failure, with the great veins distended with blood, dilatation of the right auricle and ventricle, and acute gastric erosions.

### THE EFFECTS OF COLD ON THE HUMAN BODY

The effects of exposure to low temperature have been described in detail by Burton and Edholm (1955), and much of what follows is taken from their chapter on hypothermia and resuscitation.

The body has two temperatures, external and internal. The external temperature, which is that of the skin and subcutis, varies a good deal with the environment, but the internal temperature (best measured in the rectum) is fairly constant at 37°C

* This paper was read to the Summer Meeting of the Cave Research Group of Great Britain at Brecon, on the 29th June 1963.

(98·5°F). When the body is cooled, the blood vessels of the skin and subcutis shut down, so that the circulation there is greatly reduced and the skin cools rapidly, until it approaches the temperature of the surroundings. This is one of two simple defensive reactions against cold, since the subcutis is composed of fatty tissue, which is a non-conductor of heat. The other is shivering. Shivering begins as soon as the surface is thoroughly chilled, and provides warmth to maintain the internal temperature. Much energy may be used up, and the heat production is equal to that of considerable exercise. With moderate cooling, a balance may be effected which can last for a long time.

With rapid or severe cooling the condition is otherwise. Most of the experimental work was done at Dachau, of all places, by Dr. Rascher and other disciples of Himmler (Alexander, 1945). They dressed their subjects in aviation suits and plunged them into water of between 2° and 12°C (35°–54°F). The actual temperature between these limits made little difference. The subject would immediately suffer excruciating pain and cramp and begin to shiver violently. The pain was worse if the nape of the neck was submerged. At first the rectal temperature and pulse would rise on account of the shivering, but after 5–10 minutes would begin to fall. At the same time the pain became much less. The reason for this is that the nerve endings in the skin become insensitive, once the blood supply has been cut off.

At a rectal temperature of 36°C (97°F) shivering is accompanied by rigidity of the limbs and tends to occur in spasms, but in some experiments would continue for nearly an hour. At a rectal temperature of 34°C (93°F) the pulse began to get slow, there was foaming at the mouth, and the muscular rigidity made it difficult to expel air from the lungs. By the time the pulse had fallen to 50 a minute, the fall in rectal temperature accelerated. At 31°C (88°F) there was clouding of consciousness. Only sleepy answers were given to questions. Between 30° and 29°C (86°–84°F), auricular fibrillation would set in. This is a reversible irregularity of the heart beat, and is directly due to the action of cold on the pacemaker of the heart. This usually occurred after 70–90 minutes of immersion.

Death from cardiac arrest (acute heart failure with ventricular fibrillation) occurs at rectal temperatures of between 27° and 24·2°C (80·5° and 75·5°F). It is very uncommon for people to survive at lower rectal temperatures than this, but cases have been recorded. When death occurs, the heart stops beating first, and irregular respirations may continue for another 20 minutes.

The time taken to reach the point of no return varies with the temperature of the water. Molnar (1946) in his study of shipwrecked mariners has shown that no-one survived more than two hours at a temperature of 43°F (6°C) or five hours at 55°F (13°C), but that survival for indefinite periods occurred at temperatures of over 70°F (21°C) (Fig. 1). Individual variation must however be great. Experienced swimmers (e.g. Channel swimmers) can maintain their internal temperatures at a constant level for six to fifteen hours at water temperatures of from 15°C–16·5°C (59°F–62°F). Long-distance swimmers such as these usually have a thick coating (1·5 to 3 cm.) of subcutaneous fat, which when cooled acts as an efficient insulator.

At post mortem the changes found are invariably those of acute heart failure with dry lungs and severe dilatation of the right ventricle, exactly as was described in the two cases of cavers.

*Re-warming*

When a severely cold subject is rewarmed slowly, shivering starts again, with a return of the excruciating pains first experienced. But besides this an unexpected thing happens; the rectal temperature continues to drop at the same rate for another 10–15 minutes by another 4°C (= 7°F), and so may reach dangerous levels (Fig. 2).

FIG. 1. *Duration of immersion of shipwreck survivors in ocean waters of different temperatures. Each circle represents the survival of at least one man. The curve represents the critical time—temperature relation for survival. Above the curve (one exception) survival is impossible, below possible.* (*Redrawn from Molnar*, 1946.)

FIG. 2. *Rectal and skin temperatures in two Dachau subjects immersed in water at 4·5°C (40°F) for 70 and 80 minutes respectively (arrows indicate time of removal from water), and then warmed in electric light cradles. Note the "afterdrop" of rectal temperature, following removal from water.* (*Redrawn from Rascher's graph in Alexander* 1945.)

It is this which accounts for the cases of immersion which die after they have been rescued. This phenomenon is called "after-drop" and has been clearly demonstrated by all experimenters. It is due to the cooling of the central blood by the skin, when the superficial blood vessels open up.

It is found, however, that *rapid* rewarming, as by immersion in a hot bath of water at 45°C–55°C (113°–131°F) can prevent this after-drop completely, since the superficial tissues are re-warmed so fast that they cannot cool the blood when it begins once more to flow in them. Rascher has found that resuscitation by this method is possible even when respiration has ceased and the heart has stopped beating. He has answered the objection that hot baths may not be available, by throwing water heated to 55°–60°C (131°–140°F) over the severely cooled subject, and has shown that significant heat loss can be prevented in this way for some 10–15 minutes. Rapid re-warming shortens the painful period of shivering during recovery from ten minutes to two (Behnke & Yaglou, 1951).

### PREVENTION AND TREATMENT OF CHILLING OF CAVERS

Chilling does not occur in a dry cave with moderate activity. In is only under wet conditions, when the clothes have become soaked, that the skin temperature is significantly reduced and shivering occurs. Individual tolerance to cold varies enormously. A thick layer of fat increases tolerance, and so does determination. Familiarity with one's surroundings makes them more tolerable, so that it is usually the inexperienced cavers who suffer most. Woollen clothing is better than cotton, even when wet, and retains the heat much better if it fits closely than if it is loose (Dachau, again). Once soaked, it is well to try not to change the water nearest one's skin, so that it can be warmed up, but it is often best to wring out one's underclothes, if one does not expect a second soaking soon.

Immersion suits have increased cavers' tolerance of cold and wet conditions very greatly. They are, however, difficult to get and perish easily. Wet suits (Little & Galpin, 1962) made of neoprene foam are the complete answer.

The taking of food in adequate quantities both before a wet caving trip and even after shivering has begun will delay the fall of inside temperature. If, however, the rectal temperature falls to dangerous levels, the blood sugar level rises, because the muscles are no longer able to utilize sugar. At this stage giving glucose is of no use. No drug is of any avail. Alcohol is worse than useless as it accelerates body cooling by causing the blood vessels in the skin to dilate.

Exercise is nearly always better than inaction. The heat produced by moderate exercise is equal to that produced by severe shivering. Heat loss need not be accelerated as long as the subject can keep out of the water. If exercise is not possible, close contact with the body of another person will prevent heat loss, but this is not an effective form of treatment for severe cold, as when the subject becomes torpid.

It is not generally realized how seriously ill the subject has become by the time this state of torpor is reached. Nor is it realized for what a short time some individuals are capable of tolerating a shower of cold water. In the first of the cases described death took place after one-and-a-half hours, in the second after one hour. This agrees quite well with Molnar's figures for shipwrecked mariners, and yet for the cavers the heat loss is not as great as it is with total immersion.

If after rescue the subject is still torpid, the rectal temperature below 32°C (89°F) and the pulse slow, treatment by immersion in a hot bath is called for as soon as possible. Its practical application would need to be considered afresh in each case, since the facilities which might be provided at the nearest farm house might be slender. Rubbing with towels is of no use, unless the skin has already been warmed. Blankets and hot waterbottles and dry clothes will result in rather slow re-warming, with a return of painful shivering and danger of death due to the after drop of rectal temperature. There are no substitutes for a hot bath.

### SUMMARY

Two cases of cavers dying of cold are described. One died one-and-a-half hours and the other one hour after getting drenched. They were both young and inexperienced and were not properly clad. They died of acute heart failure. The changes resulting from experimental cold immersion of human beings are described. They correspond with those experienced in these two cases. Slow re-warming of a dangerously cold subject may result in death due to the "after drop" of the inside temperature. Rapid re-warming in a bath of hot water (45°–55°C, 113°–131°F) is recommended.

The prevention and treatment of chilling of cavers is discussed in the light of this information.

### REFERENCES

Alexander, L. (1945). "Treatment of shock from prolonged exposure to cold, especially in water." Combined Intelligence Objectives Subcommittee Target No. 24, *Medical*, APO 413.

Behnke, A. R. and Yaglou C. P. (1951). "Physiological responses of men to chilling in ice water and to slow and fast re-warming." *Jour. Applied Physiol.*, **3**, 591–602.

Burton, A. C. and Edholm, O. G. (1955). "Man in a cold environment." Arnold, London. Ch. 11.

Little, W. H. and Galpin, L. S. (1962). "Anti-exposure suits and a wet-suit for caving." C.R.G. Publication No. 11, Section IV, 61–85.

Molnar, G. W. (1946). "Survival of hypothermia by men immersed in the ocean." *J.A.M.A.*, 131, 1046–1050.

## Figures and Tables

**Fig. 1. f1:**
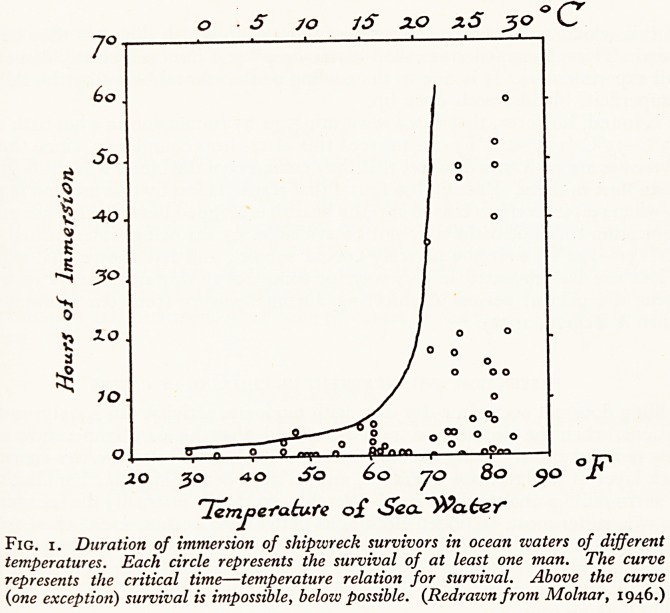


**Fig. 2. f2:**